# Does the name of a disease matter? Chinese people’s public perception of the renaming of COVID-19

**DOI:** 10.1093/pubmed/fdaf045

**Published:** 2025-04-24

**Authors:** Mengru Han, Yan Gu

**Affiliations:** Department of Chinese Language and Literature, East China Normal University, 500 Dongchuan Road, Shanghai 200241, China; Department of Psychology, University of Essex, Wivenhoe Park, Valley Road, Colchester CO4 3SQ, UK; Department of Experimental Psychology, University College London, 26 Bedford Way, London WC1H 0AP, UK

**Keywords:** health communication, public perception, naming a disease, linguistic framing, COVID-19, language and thought

## Abstract

**Background:**

On 7 December 2022, China discontinued its 3-year zero-COVID strategy, and on 26 December 2022, changed the name of COVID-19 from 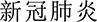 [novel coronavirus pneumonia (NCP)] to 
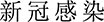
 [novel coronavirus infection (NCI)]. This study examined whether the renaming influenced public perception of COVID-19’s severity right after the change, despite the Omicron variant itself remaining *unchanged*.

**Methods:**

A survey was conducted immediately following the renaming in China. Participants were asked to directly compare the two names, and indirectly questioned about their perceptions of the virus. Responses were compared to assess whether linguistic framing with NCP or NCI influenced perceptions.

**Results:**

Direct comparisons showed that 65% of respondents (*N* = 1256) perceived the new name as less serious and frightening than the old one. However, one-third of participants did not perceive such differences, which was associated with their education level, age, and relationship status. Indirect comparisons revealed that perceived severity of COVID-19 was influenced by an interaction between wording in names and participants’ intensity of COVID-19 experience.

**Conclusions:**

Linguistic framing, personal experience, and sociodemographic factors can all influence disease perceptions during health crises. Optimizing naming strategies can reduce public anxiety and enhance health communication.

## Introduction

On 11 February 2020, the World Health Organization (WHO) officially named the coronavirus disease COVID-19. China, using a non-alphabetic writing system, referred to it as 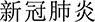 [novel coronavirus pneumonia (NCP)] until the end of 2022. On 7 December 2022, the National Health Commission of China (NHC) discontinued the zero-COVID strategy after nearly 3 years of strict COVID-19 protocols. Immediately following the drastic policy change, about 64% of the population (around 900 million) was infected, with 59 938 hospital deaths reported between 8 December 2022 and 12 January 2023.^1^^,^^2^ On 26 December 2022, the NHC renamed COVID-19 from 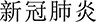 (NCP) to 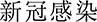 [novel coronavirus infection (NCI)], reflecting changes in disease characteristics. COVID-19 was named ‘NCP’ at the beginning of the pandemic because most patients had pneumonia symptoms. As the Omicron variant became the predominant strain, the pathogenicity decreased, resulting in fewer individuals exhibiting symptoms of pneumonia. The renaming aimed to more accurately describe the condition and mitigate public fear of COVID-19.^3^ The study investigates the impact of this renaming on public perception.

### Naming, renaming a disease, and public perception

Naming a new disease appropriately is crucial during a pandemic.^4^ The WHO emphasizes the importance of naming in medical and communication contexts,^5^ stating that it should ‘[…] minimize unnecessary negative impact of disease names on trade, travel, tourism, or animal welfare, and avoid causing offence to any cultural, social, national, regional, professional, or ethnic groups’.^5^ In the early stage of the disease spread, naming choices varied significantly across media and political discourse.^6^^,^^7^ Following its guidelines, WHO carefully named COVID-19 at the onset of global health crisis, before its official pandemic declaration.^8^

While its regulations do not explicitly address renaming established diseases or their impact on public perception, WHO has renamed diseases based on public perception, scientific recommendations, and other factors. As noted by a reviewer, monkeypox was renamed mpox in 2022.^9^ Research on how renaming diseases influences public perception remains limited. Exploring this issue could offer evidence-based guidance for effective public health communication, especially during pandemics.

### The effect of linguistic framing on people’s perception of diseases

Language influences perception, thoughts, and reasoning across domains.^10–12^ Particularly, metaphorical language can alter how diseases are perceived. For example, framing depression as a disease reduces perceived personal responsibility,^13^ while describing cancer as a ‘battle’ evokes guilt if recovery fails, unlike framing it as a ‘journey’.^14^ Such language facilitates communication and affects thinking even if not literally accurate.^15^ Linguistic framing has also been shown to promote vaccination during the COVID-19 pandemic.^16^^,^^17^

Unlike constructing a hypothetical metaphorical narrative or adjusting public health messages, renaming COVID-19 in Chinese naturalistic context only involves a critical change: replacing ‘pneumonia’ with ‘infection’. While ‘pneumonia’ conveys severity, it is unclear whether this change immediately impacts public perception. Understanding this impact can advise language use in health communication.

### Cognitive factors, embodied experience, and perception

Cognitive factors, such as effect and pragmatic reasoning, may influence perceptions of the renaming. Effective responses to ‘pneumonia’ and ‘infection’ evoked by the two names likely differ, with ‘pneumonia’ carrying more negative connotations and eliciting greater fear.^18^ If perception relies solely on the valence and arousal of wording, NCP would likely be seen as more negative, indicating greater severity and evoking more fear than NCI.

Pragmatic reasoning may also play a role. Defined as ‘the process of finding the intended meaning(s) of the given’ by inferring appropriate contexts,^19^ it suggests that speakers select frames to convey beliefs, while listeners interpret these views based on the chosen frames. Throughout the policy transition, the media extensively advocated the reasoning behind the renaming, and discussions on social media ensured that the public was aware that the renaming aimed to alleviate their concerns regarding COVID-19. If pragmatic reasoning influences views of the two names, NCI is likely seen as less serious than NCP.

Additionally, personal experience influences perception of chronic obstructive pulmonary disease, pain perception, and mental illness.^20–22^ Regarding COVID-19, individuals diagnosed with COVID-19 perceived more negative consequences, while those with infected partners reported greater personal control.^23^ However, whether embodied experience affects illness perception differently based on the disease naming or renaming remains unclear.

### This study

While naming a disease appropriately is crucial in health communication, no large-scale empirical surveys have examined how renaming affects public perception in real-life contexts. The renaming of COVID-19 during its rapid spread, widely covered by media and attracting public attention, provides an ideal case for study. We report findings from a timely survey conducted immediately after COVID-19 was renamed from ‘NCP’ to ‘NCI’ in China. We aim to reveal the impact of renaming on Chinese public perception while considering cognitive, embodied experience, and sociodemographic factors (including gender, age, education, marital status, and occupation).^24^^,^^25^

We employed both direct (within-subject) and indirect (between-subject) questions to examine perception of the renaming. First, Chinese participants directly compared the old (NCP) and new (NCI) names. If linguistic framing immediately influences thinking, the new name would be perceived as less threatening and frightening. If perception reflects changes in the virus rather than the name, participants would agree that NCP is more severe than NCI. Second, to reduce metalinguistic processing, we created indirect questions about participants’ perceptions of the virus and compared responses across participants. This assessed whether perception of COVID-19 is based on framing with NCP or NCI.

In addition to investigating the impact of language on public perception, we examined how embodied experience (personal experience with COVID-19, such as infection status, perceived severity and degree of discomfort, or knowing someone hospitalized) and sociodemographic factors affected responses.

## Method

### Participants

A total of 1256 Chinese participants (*M_age_* = 32.44 years, *SD* = 14.4; 799 females; 457 males) were randomly assigned to two conditions: NCP (the old name) and NCI (the new name). Participants, representing all provinces in China, met inclusion criteria: ≥18 years, native Chinese speakers, and residing in China. Most had a college-level education or higher. Participants with incomplete, identical responses across all items or survey completion time under 3 min were excluded (*N* = 512). The final analyses included 744 participants (Table 1).

**Table 1:** Sociodemographics of the participants included in the final analyses (*N* = 744).

Sociodemographic characteristics	*N*	*%*
Gender		
Female	490	0.66
Male	254	0.34
Age (year)	*M* = 37.8, *SD* = 15.4, 18–84 years	
Marital status		
Married	429	57.7
In a relationship	74	9.9
Single	209	28.1
Divorced	21	2.8
Widowed	11	1.5
Education level		
Primary school and below	4	0.5
Middle school	32	0.4
High school/technical secondary school/technical school	115	15.5
Junior college	95	12.8
Bachelor’s degree	369	49.6
Master’s degree and above	129	17.3
Occupation		
Students	204	27.4
Government employees/civil servants	27	3.6
Managers (including chief executives)	42	5.6
Staff (office staff)	92	12.4
Professionals (doctors/lawyers/cultural and related associate professionals/journalists/teachers)	141	19.0
Workers	29	3.9
Service and sales workers	20	2.7
Self-employed/contractors	11	1.5
Freelancers	22	3.0
Agricultural, forestry, and fishery workers	0	0
Retired	129	17.3
Temporarily unemployed	14	1.9
Others	13	1.7

### Survey design

The questionnaire included (1) direct (within-subject) and indirect (between-subject) questions about the renaming from NCP to NCI; (2) questions about participants’ experiences with COVID-19, such as infection status, timing of positive tests, underlying health conditions, and knowledge of hospitalized friends or relatives due to COVID-19; and (3) sociodemographic factors.

We created two versions of the survey, with identical structures and questions but different framing of indirect (between-subject) questions (Q1–Q5, e.g. Q1: To what extent do you think *NCP (or, in the alternative version, NCI)* affects your overall health given the current pandemic situation? 1 = Hardly any impact, 7 = Extremely severe and life-threatening). These questions used either ‘NCP’ or ‘NCI’ and appeared at the beginning of the survey. By comparing outcomes between versions, we indirectly examined whether individuals perceived the two names differently. Finally, we directly asked participants if they perceived differences between the two names through within-subject questions (Q6 and Q7). The options for all questions were randomized (see Supplement 1).

### Procedure

The survey was conducted via Tencent Survey and distributed on WeChat, a popular Chinese social media platform. Participants were invited to complete a questionnaire about COVID-19. Data collection occurred from 2 to 12 January 2023, only a week after the announcement of the name change on 26 December 2022, but the emergence and initial spread of Omicron had been >1 year. Participants were asked whether their first tested positive for COVID-19 was before 7 December 2022, when China ended its zero-COVID policy. Prior to accessing the questionnaire, all participants gave informed consent. The mean completion time for the survey was 5.6 min (*SD* = 6.97). Participants received financial compensation (a lottery ranging from 1 to 4.81 CNY). The study obtained ethical approval (ETH2223-0723).

### Data analysis

Data were analysed using R.^26^ A Chi-square goodness-of-fit test was first used to examine the distribution of participants’ responses to the direct questions regarding whether they perceived a difference between the two names, NCP and NCI (Q6 and Q7). Then generalized linear models were used to predict participants’ responses. The dependent variable was a binary response to the three options, with predictors including COVID-19-related experience such as infection status, severity, degree of discomfort, and familiarity with someone hospitalized [Hospitalized (Yes/No)], as well as sociodemographic factors (age, gender, marital status, education, cohabitation with a child, etc.).

For the indirect questions (Q1–Q5), we examined the effect of Naming Condition (NCP/NCI) on participants’ responses, controlling for their COVID-related experience and sociodemographic factors. Participants were divided into two groups: those who were infected with or were experiencing symptoms of COVID (Positive group) and those who were not (Negative group). Results were reported separately, as the Negative group only answered their estimated discomfort and expected number of fever days. The analysis for the Positive group included interactions between Condition (NCP/NCI), discomfort, and Hospitalized (Yes/No) (except Q4, where discomfort was the dependent variable itself). Since Q1 and Q2 both measured the perceived severity, we used a linear mixed-effect model with a random intercept for each participant and included Question as a control variable. For the Negative group, we replaced the discomfort with a hypothetical estimation (Q4b). For other questions, we used linear models.

## Results

Table 1 presents participants’ sociodemographic information. 73.5% (*N* = 547) had been infected with COVID, 9.1% (*N* = 68) were currently infected, and 17.3% (*N* = 129) had never been infected. Notably, under China’s zero-COVID policy until 7 December 2022, only about 8% of participants were infected before this date, meaning most were infected within a month preceding the survey. Individuals with lower education (*β* = −0.22, *P* = .039), younger age (*β* = −0.03, *P* = .004), and those with a friend or relative hospitalized due to COVID-19 (*β* = 0.57, *P* = .009) were more likely to have been infected.

### Direct perception of NCP and NCI (Q6 and Q7)

**Figure 1:**
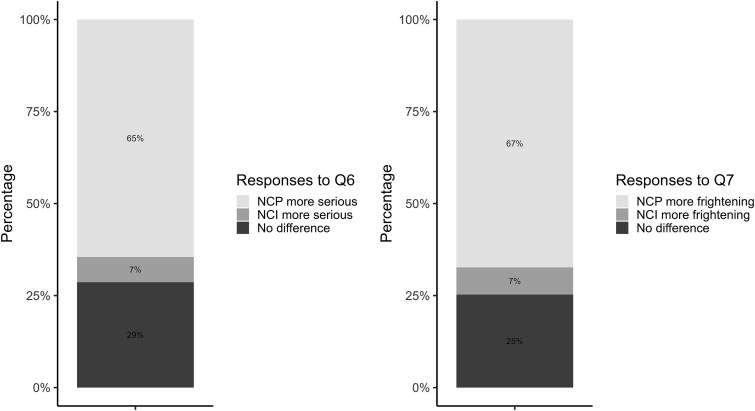
Percentages of the responses to the direct questions on the name change (Q6: On 26 December 2022, ‘NCP’ was renamed ‘NCI’. Do they sound different to you?; Q7: Which do you think sounds more frightening, ‘NCP’ or ‘NCI’?). Alt text: bar graph showing percentages of responses to questions about the name change from ‘NCP’ to ‘NCI’, including perceived differences and which name sounds more frightening.

Significant differences were observed among the three options for both Q6 (χ^2^ (2, *N* = 744) = 378.46, *P <* .001), and Q7 (χ^2^ (2, *N* = 744) = 422.81, *P <* .001), indicating that response distributions differed from chance. As Fig. 1 shows, ~65% of participants believed NCP was more severe or frightening, suggesting that the name change could indeed have immediately impacted two-thirds of participants’ perception. However, nearly 30% saw no differences in severity (29%) or fearfulness (25%). A minority believed NCI to be more severe (7%) or frightening (7%) instead. These results showed that the specific wording of the renaming, combined with the exposure to media messages, still could *not* fully account for participants’ choices of perceiving NCI as more severe/frightening or perceiving no difference between the names. We further analysed other influencing factors.

For Q6, higher education (*β* = 0.17, *P* = .049) and younger age (*β* = −0.02, *P* = .013) predicted perceiving NCP as more threatening to health, while lower education predicted the opposite (NCI as more threatening, *β* = −0.40, *P* = .009), controlling for gender, age, living with minors, marital status, COVID infection status, severity, degree of discomfort, and hospitalized (*P’s* > .05). This indicated that higher-educated and younger people were more affected by the linguistic wording in the renaming, while those with lower education were affected in the opposite direction.

For Q7, participants not in a relationship (*β* = −0.81, *P* = .026) perceived NCP as more frightening, while those with a lower education (*β* = −0.37, *P* = .016) lived with a child (*β* = 0.70, *P* = .047), and in a relationship (*β* = 1.27, *P* = .044) perceived NCI as more frightening, ceteris paribus (*P’s* > .05). In short, education and relationship status influenced perception of the renaming.

### Indirect tests of perceiving NCP and NCI (Q1–5)

*Q1 and Q2: perceived severity (General Impact on Health and Recovery Difficulty)* For the Positive group, condition of naming showed no significant main effect (see means in Table 2, *β* = 0.08, *P* = .279). However, main effects were found for degree of discomfort (*β* = 0.39, *P <* .001), age (*β* = 0.02, *P <* .001), and hospitalized (*β* = 0.49, *P <* .001), while Question and Gender were not significant (*P’s* > .05), ceteris paribus.

**Table 2:** The means (*SD*s) for the ratings of the indirect questions as a function of naming condition (NCP/NCI) (Q1–Q5).

Questions			NCP	NCI
1	General impact on health (1–7)		4.20 (1.25)	4.21 (1.26)
2	Recovery difficulty (1–7)		4.13 (1.12)	4.13 (1.09)
3	Rest time (days)		13.2 (8.63)	12.7 (8.62)
4a	Degree of discomfort (1–7)	had been infected	3.77 (8.43)	3.88 (8.54)
		being infected	4.08 (9.32)	3.94 (7.93)
4b	Estimated degree of discomfort	only for negative group	3.93 (9.09)	4.27 (9.27)
5	Expected fever days		2.95 (1.18)	3.22 (1.41)

Furthermore, significant two-way interactions occurred between Condition and Hospitalized (*β* = 1.12, *P* = .023), Condition and Degree of discomfort (*β* = 0.23, *P* = .002), and Hospitalized and Degree of discomfort (*β* = 0.20, *P* = .021). A three-way interaction was also found between the Degree of discomfort, Condition, and Hospitalized (*β* = −0.26, *P* = .027).

**Figure 2:**
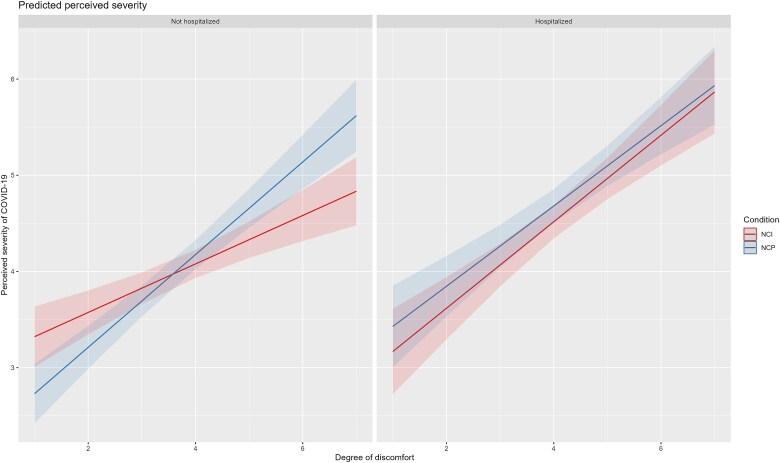
Predicted perceived severity of COVID-19 (Q1 and Q2) in the Positive group (degree of discomfort: 1 = not uncomfortable at all, 7 = intolerable).

As shown in Fig. 2, participants’ perceived severity of COVID depended on an interaction between the linguistic framing of the disease name and COVID experience. For participants without hospitalized acquaintances, those with low discomfort during their own COVID-19 infection perceived the new name as more severe than the old name (e.g. Degree of discomfort = 1, *β* = 0.59, *P* = .034) or showed no significant differences (e.g. Degree of discomfort = 2, *β* = 0.36, *P* = .088; Degree of discomfort = 3, *β* = 0.13, *P* = .579). Those with high discomfort perceived the opposite, with the old name as more severe (Degree of discomfort = 6, *β* = −0.56, *P* = .018; Degree of discomfort = 7, *β* = −0.79, *P* = .012). Among participants with hospitalized acquaintances, naming Condition did not significantly affect perceived severity (all *P*’s > .5).

As for the Negative group, the naming Condition showed no significant effect (*β* = −0.001, *P* = .95). However, estimated Degree of discomfort (*β* = 0.50, *P <* .001), Hospitalized (*β* = 0.55, *P* = .003), and Living with a child (*β* = 0.43, *P* = .018) were significant effects. No interactions between Condition and other predictors were observed (all *P*’s > .3).

**Q3-Q5:** for both the positive and negative groups, there was no significant effect of Condition or interactions between Condition and other predictors (all *P*’s > .5) in any question, ceteris paribus. Significant predictors for the Positive group were Degree of discomfort (*β* = 1.85, *P <* .001), Hospitalized (*β* = 3.00, *P <* .001), and Age (*β* = 0.10, *P <* .001) for estimated rest time. For the Negative group, the estimated Degree of discomfort (*β* = 2.39, *P <* .001) and Living with children (*β* = 3.41, *P <* .001) were significant predictors. See detailed results of Q4 and Q5 in Supplement 2.

## Discussion

### What is already known on this topic

Naming a new disease properly during a pandemic is essential.^4^ The WHO highlights that the naming should minimize unnecessary negative impacts.^4^ The renaming by the NHC aimed to more accurately reflect the virus’ characteristics and alleviate public concerns when ending the zero-COVID policy.^3^ Linguistic framing can alter how diseases are perceived,^12–14^ and promote vaccination during the COVID-19 pandemic.^16^^,^^17^

### What this study adds

Our findings have practical implications for public health communication during a pandemic. The new name appears to reduce the perceived severity and fearfulness for about two-thirds of people, but only when directly compared with the old name. When using the new name alone, its impact is mainly observed among individuals who suffered severely from COVID, while those with mild or no infection perceived little difference. To maximize the effectiveness of renaming efforts, media outlets should emphasize comparisons between the old and new names, particularly in the early stages of renaming.

The findings shed light on the effect of linguistic framing on cognition. On the one hand, a simple renaming can swiftly alter the perceptions of severity and concern for the disease for two-thirds of people. On the other hand, despite the varying valence of the two names and the intention to reduce public anxiety through renaming, not everyone agreed that NCI sounded less serious or frightening than NCP when directly comparing the two. Although NHC and the media extensively explained the reasoning behind the renaming, nearly one-third of participants still perceived no difference, and a small percentage even found NCI more severe. These findings highlight significant individual variations in how linguistic framing and media influence perceptions. Sociodemographic factors, such as education, cohabitation with a child, and marital status, played a role in shaping people’s responses. Younger and more educated individuals, who are more likely to access COVID-19 information online,^27^ were more inclined to perceive NCP as more severe and frightening than NCI. Conversely, those living with children may exhibit greater risk aversion, leading them to perceive NCI as more severe. Marital status only influenced individuals’ direct perception of the disease’s frighteningness: single participants perceived NCP as more frightening, while those in a relationship found NCI more frightening.

Additionally, we found an effect of bodily experience on perceptions of COVID-19 severity. When examined indirectly through between-subject questions using different names for COVID, a clear distinction emerged between the Positive and Negative groups. The Negative group seemed unaffected by the renaming, while the Positive group’s perceived severity between NCI and NCP depended on their embodied experiences with COVID (degree of discomfort) and indirect experiences, such as knowing someone hospitalized due to COVID. Notably, participants who experienced significant discomfort perceived NCI as less severe than NCP, whereas those with mild discomfort or in the Negative group showed no renaming effect. These results suggest that renaming a disease may not necessarily affect non-patients but can reduce the perceived frighteningness for individuals who have experienced or are currently experiencing significant suffering from a disease/problem.

### Limitations of this study

While trust in government and media may influence individuals’ COVID-19 information perceptions,^28^ this factor was not measured due to political and cultural sensitives. We could not fully disentangle the effects of public messaging during the renaming period from the name change itself. We could not account for participants’ urban or rural residence, but results remained robust when controlling for provincial economic development (see OSF analyses).

## Supplementary Material

Supplement1_Questions_fdaf045(1)

Supplement2_Analyses_Q4-Q5_fdaf045

## Data Availability

All data, analyses scripts, and output are available at: https://osf.io/s75zc/?view_only=b24d032b25c249bbbd66bebe7fa148e3

## Notes

**Mengru Han**, Associate Professor

**Yan Gu**, Assistant Professor
