# Supplementary material for: Does the name of a disease matter? Chinese people’s public perception of the renaming of COVID-19
Source: J Public Health (Oxf). 2025 Apr 24;47(3):629–36. doi: 10.1093/pubmed/fdaf045 (PMC12395955; doi:10.1093/pubmed/fdaf045)
Supplement: Supplement1_Questions_fdaf045(1) [file supplement1_questions_fdaf045(1).docx]

**Supplement 1: Questions Q1-Q7**

*Indirect questions (between-subject design)*

Q1: To what extent do you think **novel coronavirus pneumonia (or, in the alternative version, novel coronavirus infection)** affects your overall health given the current pandemic situation?

根据现在的疫情情况，您认为新冠肺炎（或在另一个版本的问卷：新冠感染）总体对身体健康有多大影响？

Options: a Likert scale of 1–7, 1 = Hardly any impact（几乎没有影响）, 7 = Extremely severe and life-threatening（影响很大，危及生命）

Q2: To what extent do you think it is easy or difficult to recover from **novel coronavirus pneumonia/ novel coronavirus infection**?

您认为新冠肺炎（或新冠感染）后恢复健康的难易程度如何？

Options: a Likert scale of 1–7, 1 = Very easy（非常容易）, 7 = Very difficult（非常难）

Q3: How many days do you consider necessary for recovery after testing negative for **novel coronavirus pneumonia/ novel coronavirus infection**?

您认为从新冠肺炎（或新冠感染）转阴后需要休息多长时间？

Select: 0-30 days

Q4a: For those who were infected and were being infected: How uncomfortable were you when you experienced the **novel coronavirus pneumonia/ novel coronavirus infection**?

您新冠肺炎（或新冠感染）后觉得有多难受？

Q4b: For those who have not been infected: How uncomfortable do you think **novel coronavirus pneumonia/ novel coronavirus infection** will be?

您觉得新冠肺炎会有多难受？

Options: using a Likert scale 1–7, 1 = Not uncomfortable at all（完全不难受）, 7 = Intolerable（无法忍受）

Q5: Only for those who have not been infected with COVID-19:

How long do you think is the expected duration of fever associated with **novel coronavirus pneumonia/ novel coronavirus infection**?

您觉得新冠肺炎（或新冠感染）大概会发烧多少天？

Select: 0–10 days

*Direct questions (within-subject design)*

Q6. On December 26, 2022, “**novel coronavirus pneumonia**” was renamed “**novel coronavirus infection**.” Do they sound different to you?

2022年12月26日，“新冠肺炎”更名为“新冠感染”，您觉得听起来有区别吗？

1. Novel coronavirus pneumonia sounds like a more severe threat to health.（听起来新冠肺炎对健康影响更严重）
2. Novel coronavirus infection sounds like a more severe threat to health.（听起来新冠感染对健康影响更严重）
3. They sound no different.（听起来两者对健康影响没有区别）

Q7. Which do you think sounds more frightening, **“novel coronavirus pneumonia”** or **“novel coronavirus infection”**?

您觉得“新冠肺炎”和“新冠感染”，哪个听起来更可怕？

1. Novel coronavirus pneumonia sounds more frightening.（“新冠肺炎”听起来更可怕）
2. Novel coronavirus infection sounds more frightening.（“新冠感染”听起来更可怕）
3. They sound no different.（听起来没有区别）
