# Supplementary material for: Does the name of a disease matter? Chinese people’s public perception of the renaming of COVID-19
Source: J Public Health (Oxf). 2025 Apr 24;47(3):629–36. doi: 10.1093/pubmed/fdaf045 (PMC12395955; doi:10.1093/pubmed/fdaf045)
Supplement: Supplement2_Analyses_Q4-Q5_fdaf045 [file supplement2_analyses_q4-q5_fdaf045.docx]

**Supplement 2: Detailed results of Q4 and Q5**

**Q4: (Expected) Degree of Discomfort**

Similar to findings for Q3, there was no significant effect of Condition or any significant interactions between Condition and other predictors (all *p*’s > 0.5) for either the Positive or the Negative group.

For the Positive group, the significant predictor was Hospitalized (*β* = 0.37, *p* < 0.001), indicating that individuals who tested positive for COVID and had a friend or relative hospitalized due to the virus were more likely to experience discomfort. For the Negative group, the significant predictors for the expected degree of discomfort were Hospitalized (*β* = 0.69, *p* = 0.002), Gender (*β* = -0.43, *p* = 0.034) and Age (*β* = -0.02, *p* = 0.009). These results show that, for individuals who had not been infected with COVID, males and younger people reported lower expected discomfort, while those with a hospitalized friend or relative reported a higher level of discomfort.

**Q5: Expected Number of Fever Days by the Negative Group**

Our analysis revealed no significant predictors for the responses to Q5 (all *p*’s > 0.5).
